# Aspirin prevents colorectal cancer by regulating the abundance of *Enterococcus cecorum* and TIGIT^+^Treg cells

**DOI:** 10.1038/s41598-024-64447-0

**Published:** 2024-06-12

**Authors:** Xiaojuan Yang, Yajuan Yan, Fengkui Wang, Jinhua Tian, Qian Cao, Miao Liu, Bin Ma, Chunxia Su, Xiangguo Duan

**Affiliations:** 1 School of Basic Medicine, Ningxia Medical University, Yinchuan, 750004 China; 2 School of Inspection, Ningxia Medical University, Yinchuan, 750004 China; 3 Department of Oncology Surgery, The First People’s Hospital of Yinchuan, Yinchuan, 750004 China; 4 The First School of Clinical Medicine, Ningxia Medical University, Yinchuan, 750004 China; 5 General Hospital of Ningxia Medical University, Yinchuan, 750004 China; 6 Department of Pathogen Biology and Immunology, School of Basic Medicine, Ningxia Medical University, Yinchuan, 750004 China

**Keywords:** Cancer, Medical research, Oncology

## Abstract

Although aspirin can reduce the incidence of colorectal cancer (CRC), there is still uncertainty about its significance as a treatment for CRC, and the mechanism of aspirin in CRC is not well understood. In this study, we used aspirin to prevent AOM/DSS-induced CRC in mice, and the anti-CRC efficacy of aspirin was assessed using haematoxylin and eosin (H&E) staining and by determining the mouse survival rate and tumour size. 16S rDNA sequencing, flow cytometry (FCM), and Western blotting were also conducted to investigate the changes in the gut microbiota, tumour immune microenvironment, and apoptotic proteins, respectively. The results demonstrated that aspirin significantly exerted anti-CRC effects in mice. According to 16S rDNA sequencing, aspirin regulated the composition of the gut microbiota and dramatically reduced the abundance of *Enterococcus cecorum*. FCM demonstrated that there were more CD155 tumour cells and CD4 + CD25 + Treg cells showed increased TIGIT levels. Moreover, increased TIGIT expression on Treg cells is associated with reduced Treg cell functionality. Importantly, the inhibition of Treg cells is accompanied by the promotion of CD19 + GL-7 + B cells, CD8 + T cells, CD4 + CCR4 + Th2 cells, and CD4 + CCR6 + Th17 cells. Overall, aspirin prevents colorectal cancer by regulating the abundance of *Enterococcus cecorum* and TIGIT + Treg cells.

## Introduction

Global data show that colorectal cancer (CRC) has the third highest incidence, followed by breast and lung cancer^1^. CRC can occur as small, benign polyps inside the colon, which subsequently become malignant^2^. Despite advancements in therapeutic options, there are few effective nonsurgical treatments for CRC^3^. Therefore, novel interventions and preventive therapies are urgently needed.

Aspirin (acetylsalicylic acid, ASA) is mostly prescribed for treating patients with cardiovascular diseases^4^. Several studies now indicate that regular long-term aspirin use can significantly reduce the overall risk of cancer^5^. In addition, the US preventive services taskforce recommends aspirin to prevent colorectal cancer^6^. Increasing evidence has shown that aspirin is an antitumour drug that affects the apoptosis, proliferation, metastasis, and senescence of tumour cells^7^. However, the role of aspirin as a possible treatment for CRC patients is unclear, and the role of aspirin and its specific mechanism in CRC are not fully understood.

In the last few years, mostly indirect evidence has shown that gut microbiota dysbiosis is associated with the occurrence and progression of CRC^8^. The gut microbiota provides essential functions for digestion and nutrient absorption, including the breakdown of indigestible carbohydrates, the synthesis of vitamins, and shaping the mucosal immune system^9^. An altered gut microbiota, such as increase in *Fusobacterium nucleatum*, *Escherichia coli,* and *Bacteroides fragilis* abundance, is observed in CRC^10^. In addition, certain probiotic bacteria, such as *Streptococcus thermophilus* and *Lactobacillus rhamnosus,* have demonstrated anticarcinogenic properties^11^. Because the specific interactions between aspirin and the gut microbiota are unknown, we wondered whether aspirin could prevent CRC by modulating the gut microbiota.

Existing data indicate that the abundance and diversity of the gut microbiota play essential roles in regulating various physiological processes, such as immunity^12^, and considerable work has been devoted to characterizing the mechanisms responsible for bacteria-mediated responses to immunity^13^. The presence of specific microorganisms is thought to be associated with certain immune responses^14^. Additionally, directly modulating the host immune response can be a powerful way to disrupt the progression of cancer^15^. However, a variety of immune cells play major roles in the immune response, and the immune cells that play key roles in the antitumour immune response include macrophages (Møs) and natural killer (NK) cells^16^, in addition to CD8 + cytotoxic T cells (CTLs) and CD4 + T cells, which can be divided into four major lineages, T-helper 1 (Th1), T-helper 2 (Th2), T-helper 17 (Th17) and T-regulatory cells (Tregs)^17^. By investigating the role and function of these immune cells in response to cancer, in this study, we revealed that changes in immune cells played a key role in aspirin-mediated regulation of the gut microbiota to prevent colorectal cancer.

Furthermore, immune checkpoints can promote or stop signals in immune cells and control their activities^18^. T-cell immunoglobulin and ITIM domain (TIGIT), also known as Vsig9 or Vstm3, is an inhibitory receptor that is predominantly expressed on activated T cells, natural killer cells, and Tregs that competes with CD226 for interactions with CD112 and CD155 (PVR)^19^. The CD155/TIGIT signalling pathway can exert immunosuppressive effects by exacerbating exhaustion^20^, resulting in tumour immune evasion. Here, we show that aspirin could prevent colorectal cancer via the gut microbiota-TIGIT^+^Treg cell axis. These results provide important insights into the role of aspirin in CRC.

## Materials and methods

### Materials

Aspirin was purchased from the American Sigma Company, and azoxymethane and dextran sulfate sodium salt were purchased from Sigma Aldrich (Shanghai) Trading Co., Ltd. (Shanghai, China). Specific-pathogen-free (SPF)-grade AIN-93G feed was purchased from XiaoShu YouTai Biotechnology Co., Ltd. (Beijing, China).

### Mice

Male C57BL/6 J mice (aged 6 weeks, bodyweight 18–22 g) were purchased from Beijing Huafukang Bioscience, Beijing, China, and fed under specific pathogen-free conditions. The mice were divided into three groups with 18 mice in each group: the normal control (NC) group, the CRC control (RC) group, and the aspirin intervention group with CRC (RA). The NC group and RC group were fed specific-pathogen-free (SPF)-grade AIN-93G normal feed. The RA group was fed specific-pathogen-free (SPF)-grade AIN-93G fodder; aspirin was pre-administered at a dose of 400 mg/kg fodder to these mice, and was continuously fed for 13 weeks. Fodder was purchased from XiaoShu YouTai (Beijing) Biotechnology Co., Ltd. (Beijing, China), and fresh feed was added every two days. The mouse model of CRC was established using the azoxymethane/dextran sulfate sodium salt (AOM/DSS) method. Specifically, mice were given a single intraperitoneal injection of azoxymethane (AOM; Cat. #A5486; Sigma‒Aldrich (Shanghai) Trading Co. Ltd., 10 mg/kg body weight) during the first week. Then, each group of mice was given 2% dextran sulfate sodium salt (DSS; Cat. #42867; Sigma‒Aldrich (Shanghai) Trading Co., Ltd.) and allowed to drink normal water for 14 days; this process was repeated for three cycles.All the experimental procedures were conducted in strict accordance with the operating procedures of the Animal Experimental Center of Ningxia Medical University (Yinchuan, China; approval number: IACUC-NYLAC-2019–023). All operations were performed with the aim of minimizing the suffering of mice.

### Haematoxylin and eosin (H&E) staining

Mice were sacrificed by cervical dislocation at 13 weeks and then fully disinfected by soaking in 75% alcohol for 10 min. Next, the colon and rectum were collected, rinsed in phosphate-buffered saline (PBS), and fixed in 4% neutral-buffered paraformaldehyde for 48 h at 4 °C. Afterwards, the tissues were embedded in paraffin and processed for H&E staining, and tissue pathology was observed under a microscope.

### Immune cell isolation

Mice were sacrificed by cervical dislocation, and the spleen was dissected from surrounding tissues and processed for flow cytometry analysis. The spleen was mechanically homogenized in PBS, and the resulting preparations were filtered through 300 mesh filters and superimposed on lymphocyte separation fluid at 2000 rpm for 20 min. The mononuclear cells thus obtained were transferred to fresh tubes and then centrifuged at 1500 rpm for 10 min. Finally, the cell concentration was adjusted for flow cytometry analysis.

### Flow cytometry

The cells were resuspended in PBS and then incubated at 4 °C with monoclonal antibodies, and a BD FACSCelesta™ flow cytometer (BD Biosciences, Franklin Lakes, USA) was used to detect the cells. The antibodies we used as follow: V450-conjugated anti-CD11b, BV650-conjugated anti-F4/80, FITC-conjugated anti-CD4, APC-conjugated anti-CD25, FITC-conjugated anti-CD19, AF647-conjugated anti-GL-7, BV421-conjugated anti-TIGIT, PE-CF594-conjugated anti-CD8, BV510-conjugated anti-CXCR3, BV650-conjugated anti-CCR6, PerCP-Cy5.5-conjugated anti-CXCR5 and BV650-conjugated anti-IL-10, were purchased from BD Biosciences (Franklin Lakes, USA). APC-conjugated anti-CD194 (CCR4), FITC-conjugated anti-NK-1.1(CD161), APC-conjugated anti-perforin and PE-conjugated anti-CD155, were purchased from BioLegend (San Diego, USA). PE-conjugated anti-granzyme B was purchased from eBioscience (Carlsbad, USA).

For surface antibody staining, 0.5 µL of antibody was directly added to 100 µL of cells, but intracellular antibodies were incubated with eBioscience Foxp3/Transcription Factor Staining Buffer (eBioscience, Carlsbad, USA) for 25 min before 1 µL of the antibody was added to cells.

### Faecal collection and 16S rDNA sequencing

Mouse faecal samples were collected at the 4th, 7th, 10th, and 13th weeks. The collected faecal samples were quickly put into liquid nitrogen and then transferred to a − 80 °C freezer after 48 h. Sequencing of the gut microbiota was completed by NovoGene Company (China, Beijing), and the data were analysed via the NovoMagic platform. The following steps were performed: extraction of genomic DNA, amplicon generation, PCR product quantification and qualification, library preparation, and sequencing.

The Illumina sequencing raw data were uploaded to NCBI, BioProject: https://www.ncbi.nlm.nih.gov/bioproject/PRJNA669487.

### Western blotting

Protein was extracted from mouse tissue using a protein extraction kit (KeyGen Biotech Co., Ltd., Nanjing, China). Protein concentrations were quantified using a bicinchoninic acid assay kit (KeyGen Biotech Co. Ltd., Nanjing, China). Thirty micrograms of total protein was electrophoresed through 10% sodium dodecyl sulfate polyacrylamide gels, followed by transfer onto polyvinylidene fluoride membranes (Millipore, USA). The membranes were blocked in 5% nonfat skim milk for 2 h and then incubated with primary antibodies at 4 °C overnight. The secondary antibodies were conjugated to horseradish peroxidase (Abbkine, China), and the signals were detected using an enhanced chemiluminescence kit (Advansta, England). The resulting images were analysed via ImageJ 6.0 software. The anti-GAPDH antibody was used as a control for whole-cell lysates. According to the purpose of the experiment, blots cut prior to hybridisation with antibodies according to the molecular weight of the antibody.We have provided images showing full length membranes, with membrane edges visible in Supplementary Fig. S2.

### Statistical analysis

Continuous variables are expressed as the median and range or as the mean ± standard deviation. Analysis of variance was used for statistical comparisons. All the statistical analyses were performed using SPSS 23.0, and two-sided *p* values < 0.05 were considered to indicate statistical significance.

### Statement

We confirm that the animal experiments in this study were conducted in accordance with the ARRIVE guidelines and that all the experiments were performed in accordance with relevant guidelines and regulations. We declare that our study was reviewed and approved by the Institutional Animal Care and Use Committee of Ningxia Medical University (approval number: IACUC-NYLAC-2019-023).

## Results

### The antitumour effects of aspirin in a CRC mouse model

**Figure 1:**
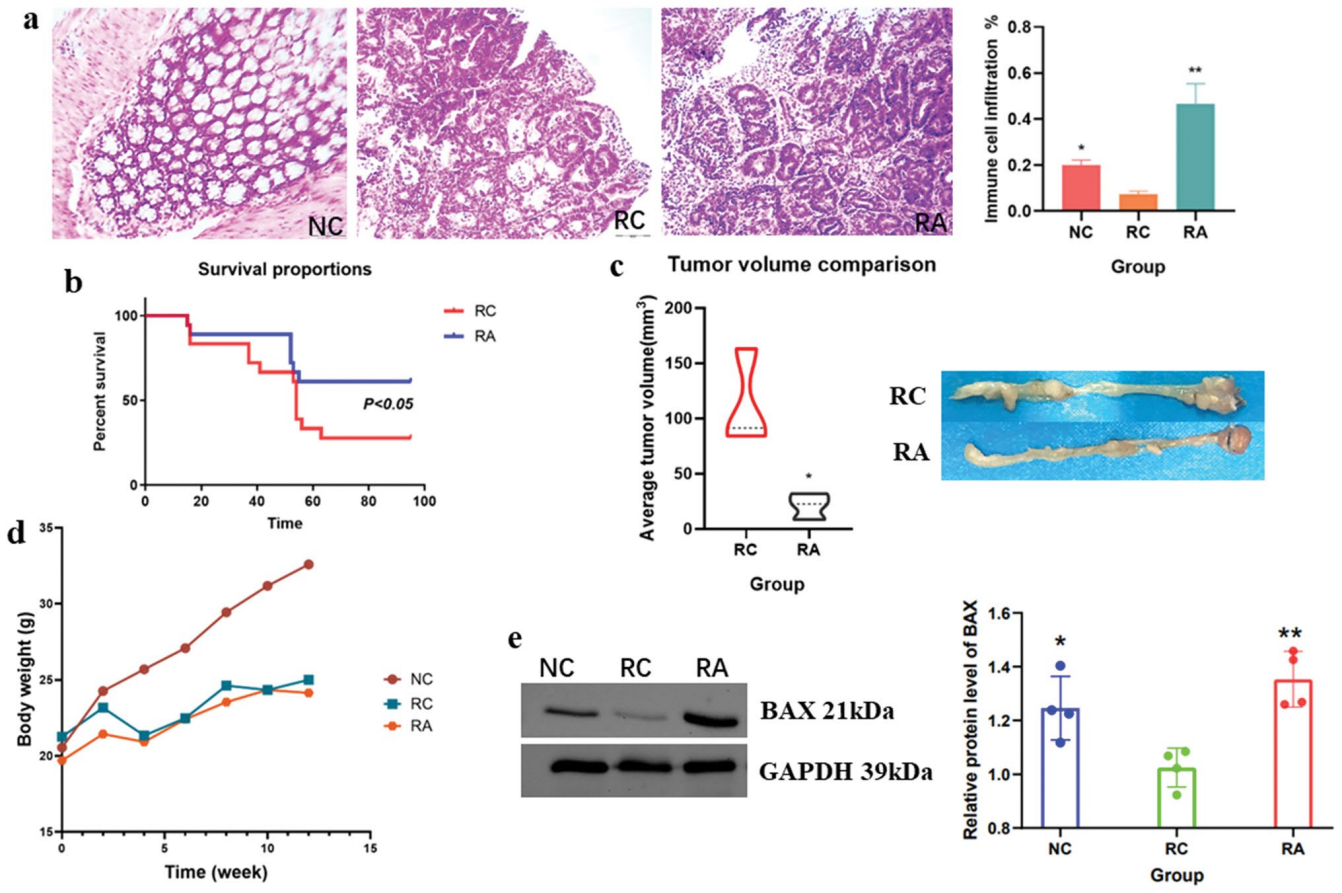
Antitumour effects of aspirin in a CRC mouse model. (**a**) Haematoxylin and eosin staining of colorectal tissue from mice. Scale bar: 50 um. n = 3. (**b**) Survival rate of CRC mice. (**c**) Comparison of the tumour volume. n = 3. (**d**) Body weight of mice during the experiment. (**e**) Protein was extracted from mouse tumour tissue for Western blotting to determined BAX expression (original blots/gels are presented in Supplementary Fig. S2). n = 4. *p < 0.05, **p < 0.01, ***p < 0.001 compared to RC.

To test our hypothesis that aspirin might function as a tumour suppressor in colorectal tumorigenesis, we utilized AOM/DSS-induced CRC mice and evaluated the antitumour effects of aspirin on CRC mice at week thirteen. We observed that aspirin significantly reduced the degree of colorectal injury in CRC mice and promoted the infiltration of inflammatory cells into CRC tissues (Fig. 1a). Survival analysis and tumour volume comparison of CRC mice showed that aspirin effectively promoted survival and reduced tumour volume in CRC mice (Fig. 1b,c). Furthermore, we observed that aspirin had no effect on the body weight of CRC mice (Fig. 1d). In our previous study, we found that aspirin can promote the expression of the pro-apoptotic protein BAX in colorectal cancer cells^21^. Here, we analyzed the expression of BAX in CRC mouse tumour tissues and found that aspirin significantly increased the expression of the BAX protein in CRC tissues, consistent with in vitro results (Fig. 1e). These results suggest that aspirin improves colorectal cancer prognosis.

### Aspirin altered the gut microbial composition in a CRC mouse model

**Figure 2:**
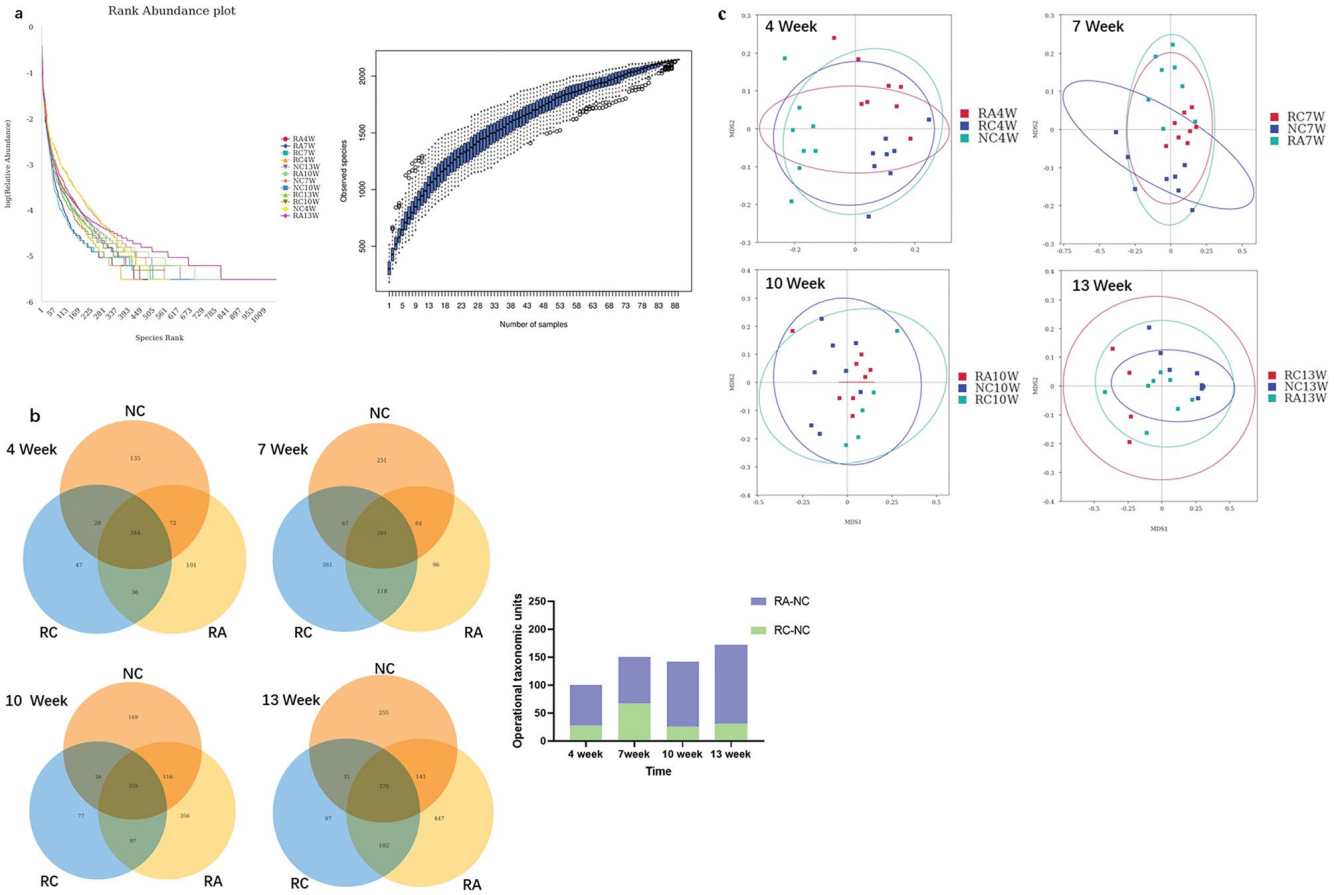
Aspirin altered the gut microbial composition in the CRC mouse model. (**a**) Rank abundance and species accumulation boxplot. The rank abundance was obtained by plotting the ranking number of OTUs as the horizontal coordinate and the relative abundance as the vertical coordinate. A species accumulation boxplot was generated for the biodiversity and community survey. (**b**) Venn diagram at weeks 4, 7, 10, and 13. A Venn diagram was generated based on the OTU analysis. (**c**) Nontetric multidimensional scaling at weeks 4, 7, 10, and 13. NMDS is a nonlinear model based on the Bray‒Curtis distance for analysis.

Next, we compared the gut microbiota compositions of different mouse models to determine whether the anti-CRC effect of aspirin was related to the gut microbiota. Boxplots of the rank abundance and species accumulation indicate that the sequencing samples were qualified and that the sequencing data were reasonable (Fig. 2a). The Venn diagram clearly shows that the number of OTUs shared by the RA and NC groups was significantly greater than that shared by the RC group at different time points (Fig. 2b). Non-Metric Multi-Dimensional Scaling (NMDS) analysis revealed that as the duration of aspirin intervention increased, the samples in the RA group gradually became more similar to those in the NC group, while the opposite trend was observed for the RC group (Fig. 2c). These findings suggest that the gut microbiota was altered in CRC mice, while aspirin effectively altered the gut microbial composition in a CRC mouse model.

### Aspirin reduced the abundance of *Enterococcus cecorum* in the CRC mouse model

**Figure 3:**
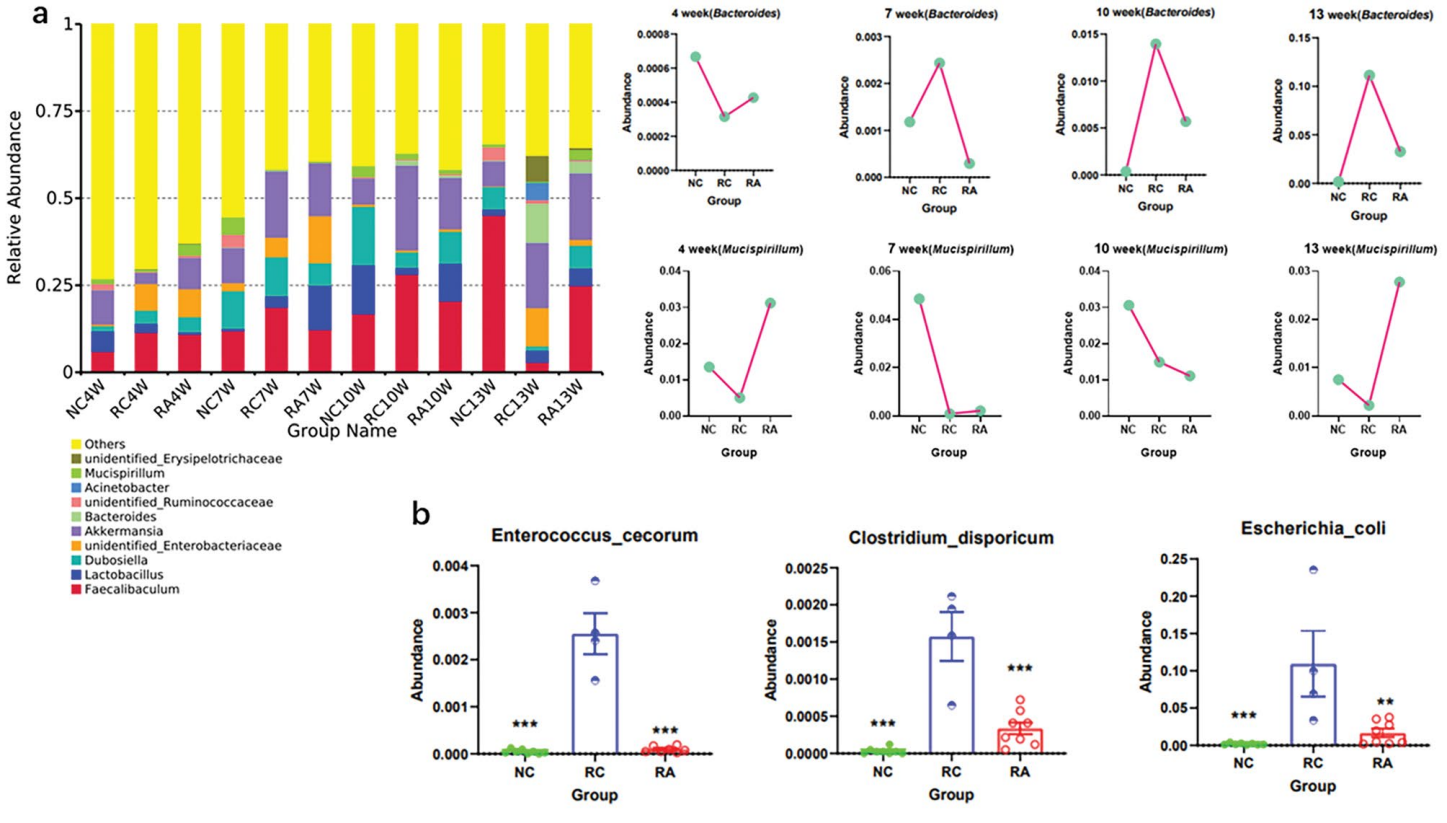
Aspirin reduces the abundance of *Enterococcus cecorum* in a CRC mouse model. (**a**) Relative abundance histogram at the genus levels. To determine the relative abundances, the number of tags corresponding to a species in a sample at a certain classification level was divided by the total number of tags corresponding to the OTUs clustered in the sample. (**b**) MetaStat diagrams of the species levels at week 13. *p < 0.05, **p < 0.01, ***p < 0.001 compared to RC.

To determine which species are affected by aspirin, we analysed the changes in bacteria at the species level. At the phylum level, the relative abundance histogram showed that the RC group had a reduced abundance of *Deferribacteres,* and the RA group had an increased abundance of *Acidobacteria* (see Supplementary Fig. S1 online). At the genus level, the relative abundance histogram indicated that the RC group had an increased abundance of *Bacteroides* and a decreased abundance of *Mucispirillum*, while the opposite was true for the RA group (Fig. 3a). We then analysed the differences in the species levels at week 13 by using the MetaStat method and identified three species that exhibited significant differences: *Enterococcus cecorum*, *Escherichia coli*, and *Clostridium disporicum*. The difference was more significant for *Enterococcus cecorum* and *Clostridium disporicum*, but the abundance of *Enterococcus cecorum* was higher (Fig. 3b). Overall, we report here that aspirin reduced the abundance of *Enterococcus cecorum* in a CRC mouse model to exert antitumour effects.

### Aspirin decreases CD4 + CD25 + Treg cells and activates CD19 + GL-7 + B cells, CD8 + T cells, CD4 + CCR4 + Th2 cells, and CD4 + CCR6 + Th17 cells

**Figure 4:**
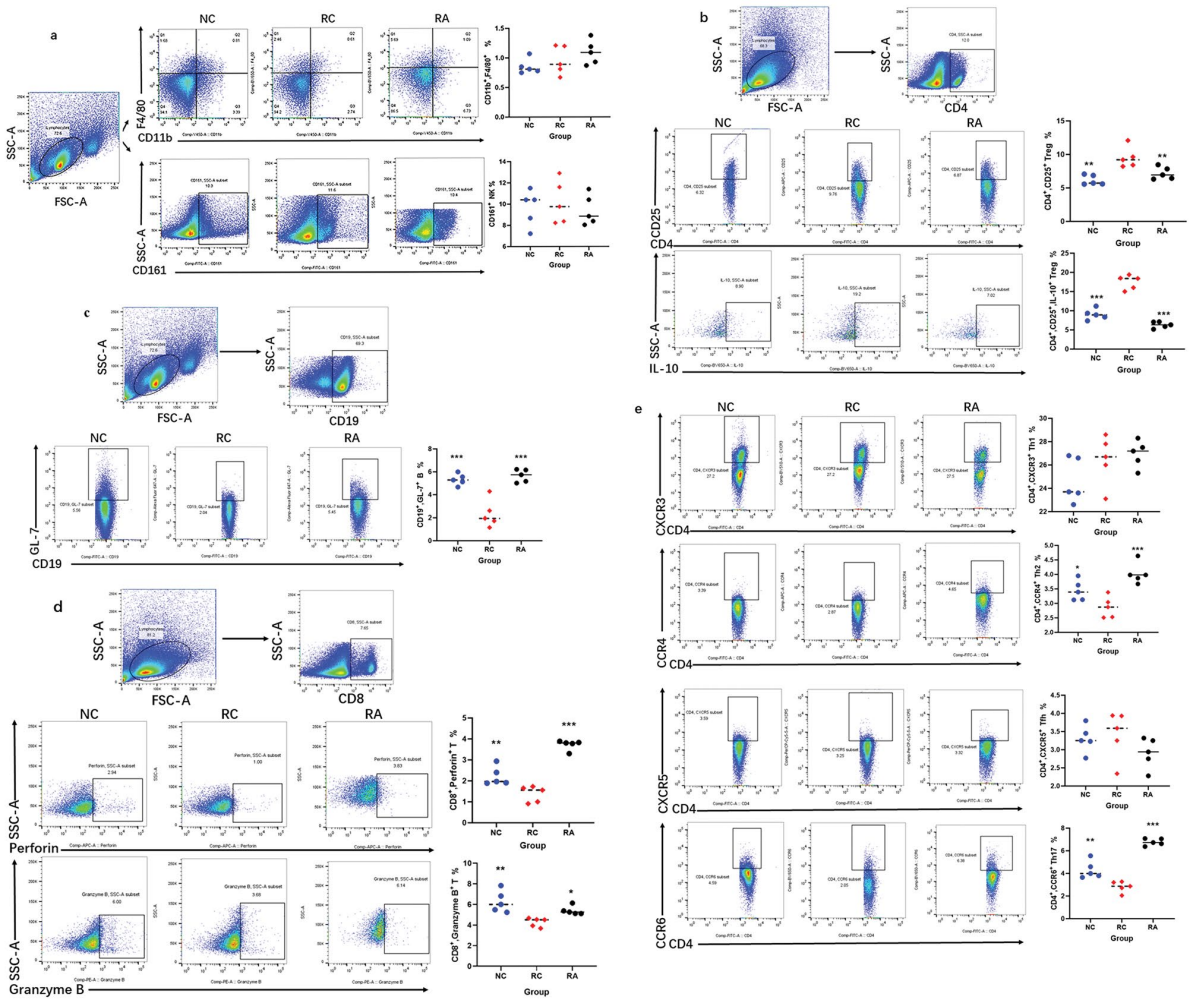
Aspirin decreases CD4 + CD25 + Treg cells and activates CD19 + GL-7 + B cells, CD8 + T cells, CD4 + CCR4 + Th2 cells, and CD4 + CCR6 + Th17 cells. Representative dot plots showing the flow cytometry gating strategy used for CD11b + F4/80 + Møs and CD161 + NK cells (**a**), CD4 + CD25 + Treg cells (**b**), CD19 + GL-7 + B cells, Perforin + CD8 + T cells, granzyme B + CD8 + T cells, CD4 + CXCR3 + Th1 cells, CD4 + CCR4 + Th2 cells, CD4 + CXCR5 + Tfh cells and CD4 + CCR6 + Th17 cells (**c**–**e**) in the mouse spleen. *p < 0.05, **p < 0.01, ***p < 0.001 compared to RC.

To confirm the essential role of the gut microbiota in immunity and to further explore the mechanism of action of aspirin, we used FCM to analyse the changes in immune cells in the mouse model. Aspirin had no effect on the natural immune cells CD11b + F4/80 + Møs or CD161 + NK cells (Fig. 4a). Treg cells are involved in tumour immune escape, and the inhibition of Treg cells is the main means of promoting antitumour immunity. Our results showed that aspirin effectively inhibited CD4 + CD25 + Treg cells and secreted IL-10 compared to those in the RC group (Fig. 4b). These findings suggest that aspirin can promote antitumour immune responses by decreasing Treg cell abundance.

To determine which cell populations are affected by aspirin through the inhibition of Treg cells, we analysed B cells, CD8 + T cells, and effector T cells. The results showed that aspirin effectively increased the ratio of CD19 + GL-7 + B cells, Perforin + CD8 + T cells, granzyme B + CD8 + T cells, CD4 + CCR4 + Th2 cells, and CD4 + CCR6 + Th17 cells (Fig. 4c–e). These results indicate that the regulatory effect of aspirin on the gut microbiota is accompanied by changes in the immune microenvironment. Aspirin administration decreases the abundance of CD4 + CD25 + Treg cells and activates CD19 + GL-7 + B cells, CD8 + T cells, CD4 + CCR4 + Th2 cells, and CD4 + CCR6 + Th17 cells.

### Aspirin downregulates the expression of TIGIT on Treg cells

**Figure 5:**
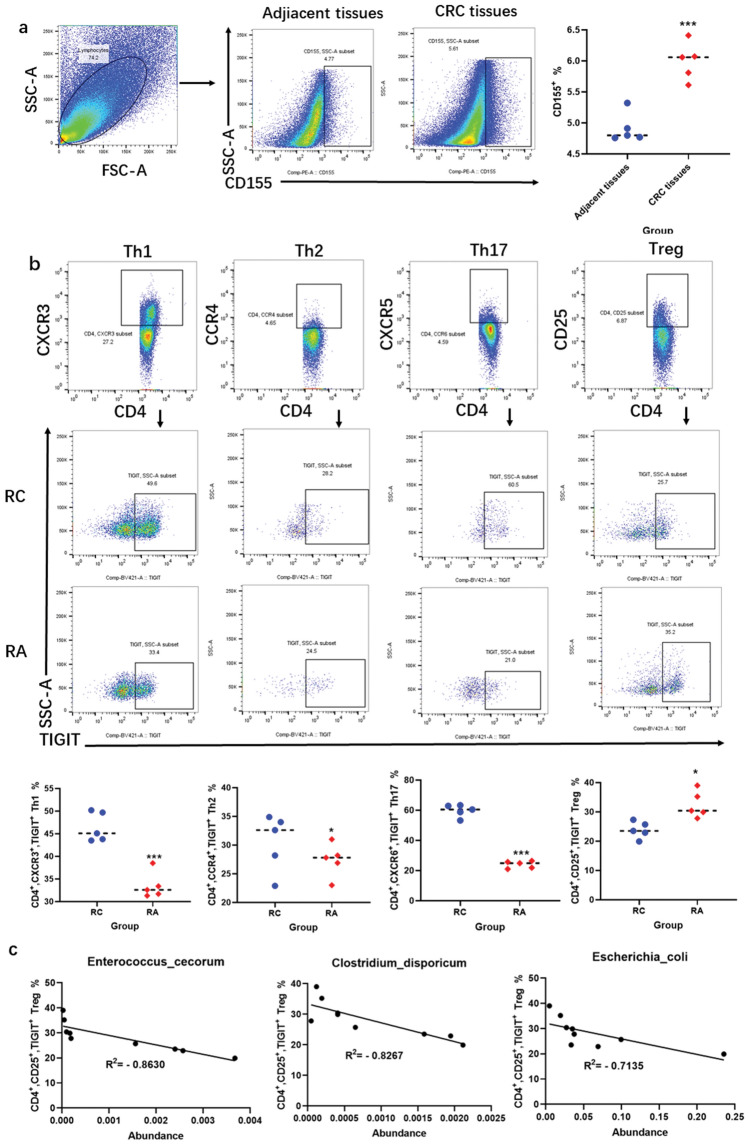
Aspirin downregulates the expression of TIGIT on Treg cells. (**a**,**b**) Representative dot plots showing the flow cytometry gating strategy used for CD155-expressing tumour tissue and adjacent tissue samples and for TIGIT-expressing T cells. (**c**) Correlation analysis between the expression of TIGIT on Treg cells and the abundance of *Enterococcus cecorum*, *Clostridium disporicum*, and *Escherichia coli*. *p < 0.05, **p < 0.01, ***p < 0.001 compared to RC.

Treg cell-mediated immune escape has been reported to be associated with TIGIT. To explore whether the suppressive effect of aspirin on Treg cells is attributed to the expression of TIGIT, we examined the expression of TIGIT and its ligand CD155. Compared with that in adjacent tissues, the expression of CD155 in cancer tissues from CRC mice was significantly increased. These results revealed that the high expression of CD155 was related to CRC (Fig. 5a). We further compared TIGIT expression on T cells and found that, compared with that in the RC group, aspirin could effectively upregulate the expression of TIGIT on CD4 + CD25 + Treg cells and inhibit the expression of TIGIT on effector T cells, including CD4 + CXCR3 + Th1, CD4 + CCR4 + Th2 and CD4 + CCR6 + Th17 cells (Fig. 5b). Correlation analysis showed that there was a negative correlation between the expression of TIGIT on Treg cells and the abundance of *Enterococcus cecorum*, *Clostridium disporicum*, and *Escherichia coli* (Fig. 5c)*.* These results indicate that aspirin can prevent immune escape and promote the antitumour immune response by increasing TIGIT expression on Treg cells.

## Discussion

The negative correlation between the “omnipotent medicine” aspirin and cancer has been explored by researchers for many years. However, there are still controversies regarding the anticancer properties of aspirin^22^, and numerous clinical trials have been launched to establish its clinical use against CRC^23^. There is no doubt that aspirin, as one of the most commonly used drugs in the world, has proven to be a life-saving agent for the prevention of cardiovascular diseases^24^. “Old trees sprout and open up new fields”, and aspirin has gradually become a hot topic for the primary prevention of cancer and as an adjuvant treatment. Unfortunately, due to the lack of sufficient pharmacological evidence on the pharmacodynamics of aspirin in the treatment of cancer patients and its ability to significantly prolong the survival of cancer patients, currently, aspirin cannot be used as an alternative drug for any cancer treatment.

However, aspirin is not the only noncancer drug that has been proven to have anticancer effects, and several successful examples, such as metformin, digoxin, and thalidomide, all show promising potential as noncancer drugs that can be repositioned to prevent or treat cancers^25^. We believe that with continuous in-depth research, the future of aspirin as an antitumour agent is promising. Considering the current controversy about aspirin and its underlying mechanism of action, in the present study, we aimed to provide a theoretical basis and experimental data on the effect and mechanism of action of aspirin in CRC.

The gut microbiota-TIGIT + Treg cell axis is our explanation of the mechanism by which aspirin affects CRC; this finding has important scientific significance. In 2015, two blockbuster papers published in Science officially ignited the study of the relationship between the gut microbiota and immunotherapy. Two teams in the United States and France have shown that the gut microbiota plays a decisive role in the effectiveness of PD-1 immunotherapy in mouse models. In recent years, several studies have shown that the microenvironment of the gut microbiota can affect the therapeutic efficacy of cancer treatment. Several articles in top journals, such as Nature and Science, have reported that microbiota in the human intestinal tract may have a direct impact on immunotherapy efficacy in cancer patients^26^. In view of these findings, we investigated whether aspirin can inhibit colorectal cancer through the gut microbiota-immune cell axis.

The gut microbiota is a complex territory of microorganisms^27^, and it is increasingly apparent that gut dysbiosis alters the physiological function of the host, leading to the occurrence of diseases^28^. There are numerous studies that emphasize the important role of the gut microbiota in cancer treatment. Some kinds of bacteria play important roles in the antitumour effect of tumour therapy, while other kinds of bacteria reduce the efficacy of tumour drugs through different mechanisms^29^. Strategies to rebalance these harmful fluctuations have been shown to be effective in the treatment of these pathologies^30^. Our study showed that *Escherichia coli*, *Enterococcus cecorum* and *Clostridium disporicum* are the driving factors of CRC, and aspirin can achieve anti-CRC effects by reducing the abundance of these three bacteria. In recent years, a functional network of connections has emerged among the gut microbiota and the immune system^31^. Studies using mouse models of various inflammatory conditions have revealed that the gut microbiota can regulate both innate and adaptive immunity^32^. With the emergence of a series of studies, an increasing number of individuals support the view that there is a "microbiota-immunity-disease" axis^33^. Our results showed that the regulatory effect of aspirin on the gut microbiota was accompanied by effects on Treg cells, B cells, CD8 + T cells, effector T cells, and Treg-centred immune cells; these effects played an important role in the anti-CRC effect of aspirin.

Importantly, a large proportion of immune cells that play a role in tumours are regulated by immune checkpoints. At present, TIGIT is considered to be one of the most promising potential targets^34^. A variety of evidence supports the idea that TIGIT plays a key role in limiting tumour adaptive immunity and innate immunity^35^. To further explore the mechanism of action of aspirin, we analysed the changes in TIGIT and its ligand CD155. The results showed that CD155 was highly expressed in CRC tumour tissues, the expression of TIGIT on Treg cells was significantly increased, and the expression of TIGIT on effector T cells was significantly decreased. These results indicated that aspirin inhibits TIGIT expression on Treg cells by regulating the gut microbiota. On the one hand, high expression of TIGIT reduces the functionality of Treg cells and reduces their immunosuppressive effect, which promotes the antitumour effect of other immune cells in the CRC immune microenvironment. On the other hand, Treg cells highly express TIGIT, which combines with CD155 expressed on tumour cells, thus preventing the binding of TIGIT expressed on effector T cells to CD155; these effects prevent immune escape and exert an antitumour effect.

There are several limitations to our study. First, from our perspective, the most probable mechanism of action of aspirin improving CRC is through regulation of the gut microbiota and immune microenvironment. However, it is still uncertain how does aspirin affect the gut microbiota by regulating immune microenvironment. Therefore, further research is needed. Second, the evidence we provide all comes from animal and cell experiments but not from humans, and as others have pointed out, evidence from human specimens is also important. Notably, the dose of aspirin and how long it will take to exert its effects are also of concern.

## Conclusions

**Figure 6:**
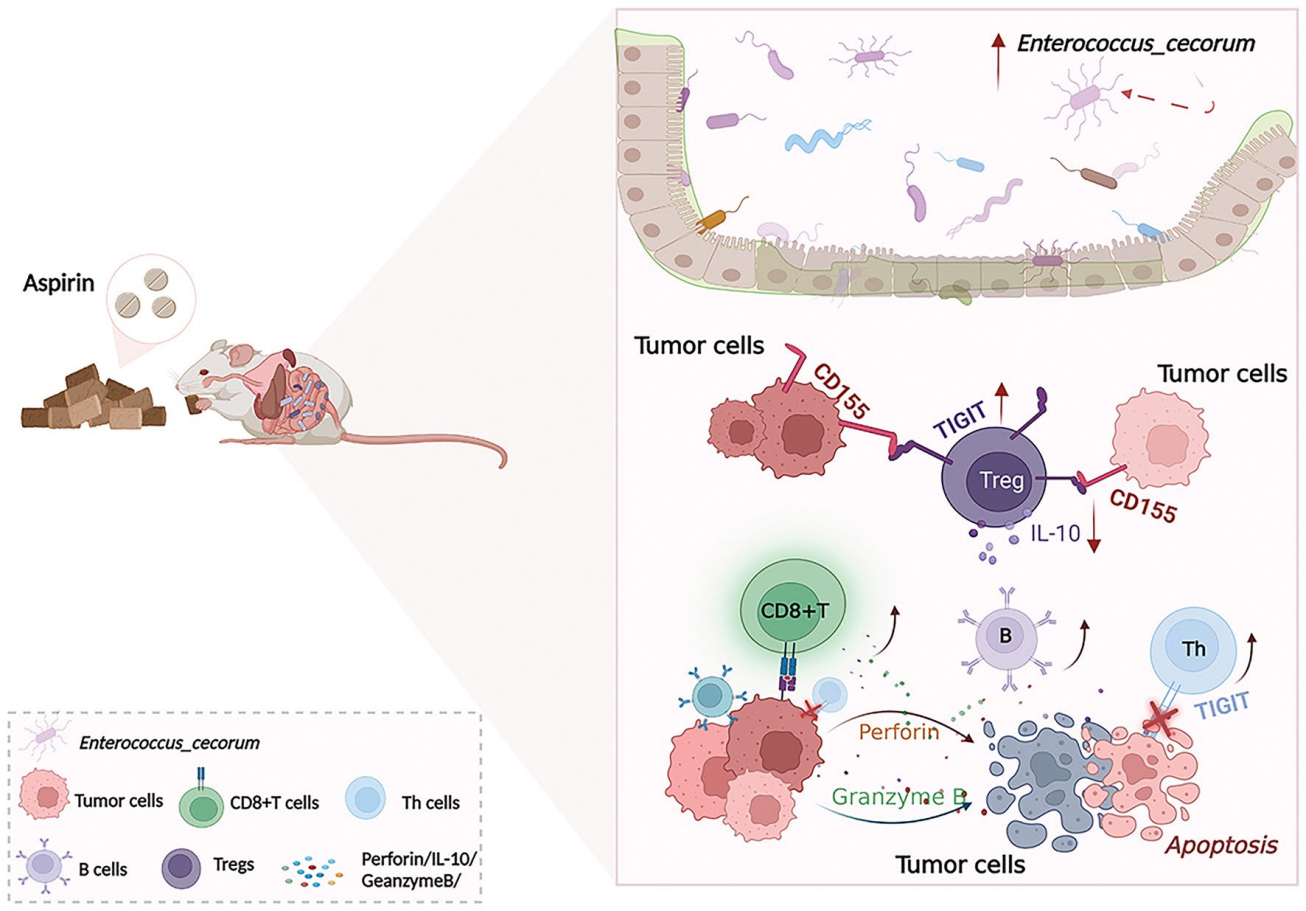
Schematic diagram of the function of aspirin in CRC. Aspirin can prevent colorectal cancer by regulating the abundance of *Enterococcus cecorum* and TIGIT + Treg cells.

In conclusion, we showed that aspirin can prevent colorectal cancer by regulating the abundance of *Enterococcus cecorum* and TIGIT + Treg cells; therefore, we believe that aspirin has a value in the treatment of CRC, and further research into aspirin and CRC would be valuable (Fig. 6).

### Supplementary Information

Supplementary Information.

Supplementary Figures.

## Data Availability

The Illumina sequencing raw data were uploaded to NCBI, BioProject: https://www.ncbi.nlm.nih.gov/bioproject/PRJNA669487. All relevant data and materials can be obtained from the first author and corresponding author.
