# Supplementary material for: Aspirin prevents colorectal cancer by regulating the abundance of Enterococcus cecorum and TIGIT+Treg cells
Source: Sci Rep. 2024 Jun 12;14:13592. doi: 10.1038/s41598-024-64447-0 (PMC11169407; doi:10.1038/s41598-024-64447-0)

Images of the original western blots

1.The following image is the western blot analysis shown in Fig. 1e

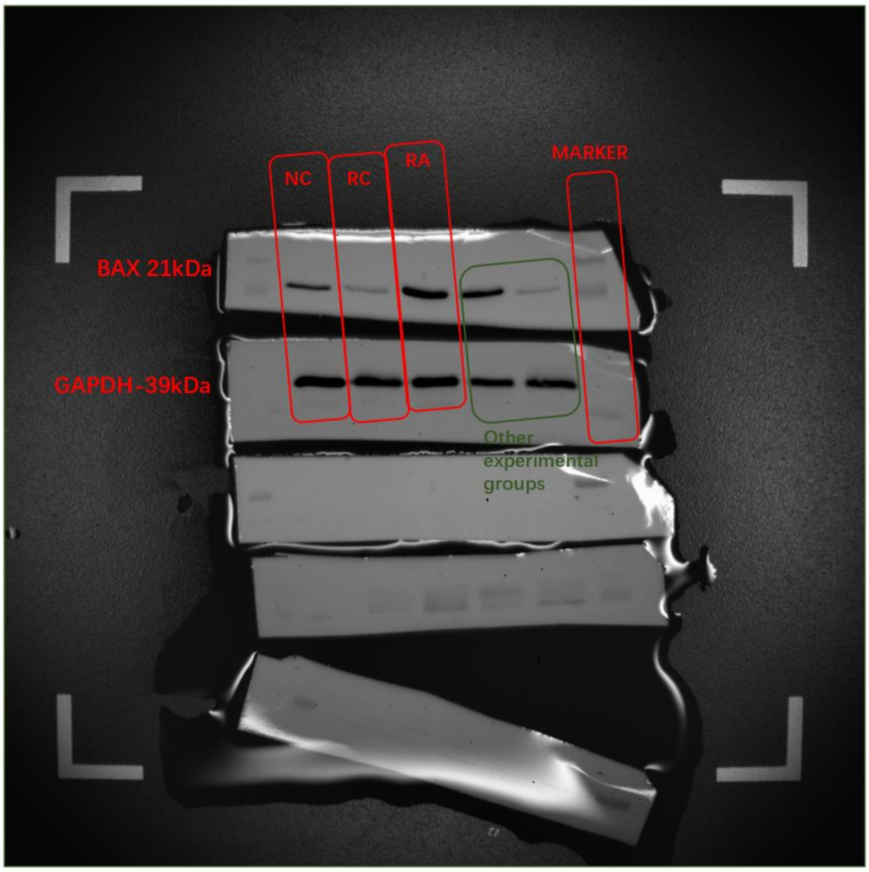

Supplement: Supplementary file 1 — Supplementary Information. [file 41598_2024_64447_MOESM1_ESM.pdf]
