# Supplementary material for: Aspirin prevents colorectal cancer by regulating the abundance of Enterococcus cecorum and TIGIT+Treg cells
Source: Sci Rep. 2024 Jun 12;14:13592. doi: 10.1038/s41598-024-64447-0 (PMC11169407; doi:10.1038/s41598-024-64447-0)
Supplement: Supplementary file 2 — Supplementary Figures. [file 41598_2024_64447_MOESM2_ESM.pdf]

Aspirin prevents colorectal cancer by regulating the abundance of *Enterococcus cecorum* and TIGIT$^+$Treg cells

Xiaojuan Yang[1], Yajuan Yan[1], Fengkui Wang[5] , Jinhua Tian[4], Qian Cao[4], Miao Liu[4], Bin Ma[3*], Chunxia Su[1*], Xiangguo Duan[2*]

1.School of Basic Medicine, Ningxia Medical University, Yinchuan 750004, China
2. School of Inspection, Ningxia Medical University, Yinchuan 750004, China.
3. Department of Oncology Surgery, The First People's Hospital of Yinchuan, Yinchuan 750004, China
4. The First School of Clinical Medicine, Ningxia Medical University, Yinchuan, 750004, China;
5.General Hospital of Ningxia Medical University, Yinchuan, 750004, China;
Correspondence*
Bin Ma, Department of Oncology Surgery, The First People's Hospital of Yinchuan, Yinchuan 750004, China. Email: 407664812@qq.com
Su Chunxia, Department of Pathogen Biology and Immunology, School of Basic Medicine, Ningxia Medical University, Yinchuan 750004, China. Email: 1651085195@qq.com
Duan Xiangguo, School of Inspection, Ningxia Medical University, Yinchuan 750004, China. Email: 2455281549@qq.com; duanxiangguo@nxmu.edu.cn

Supplementary Fig. S1

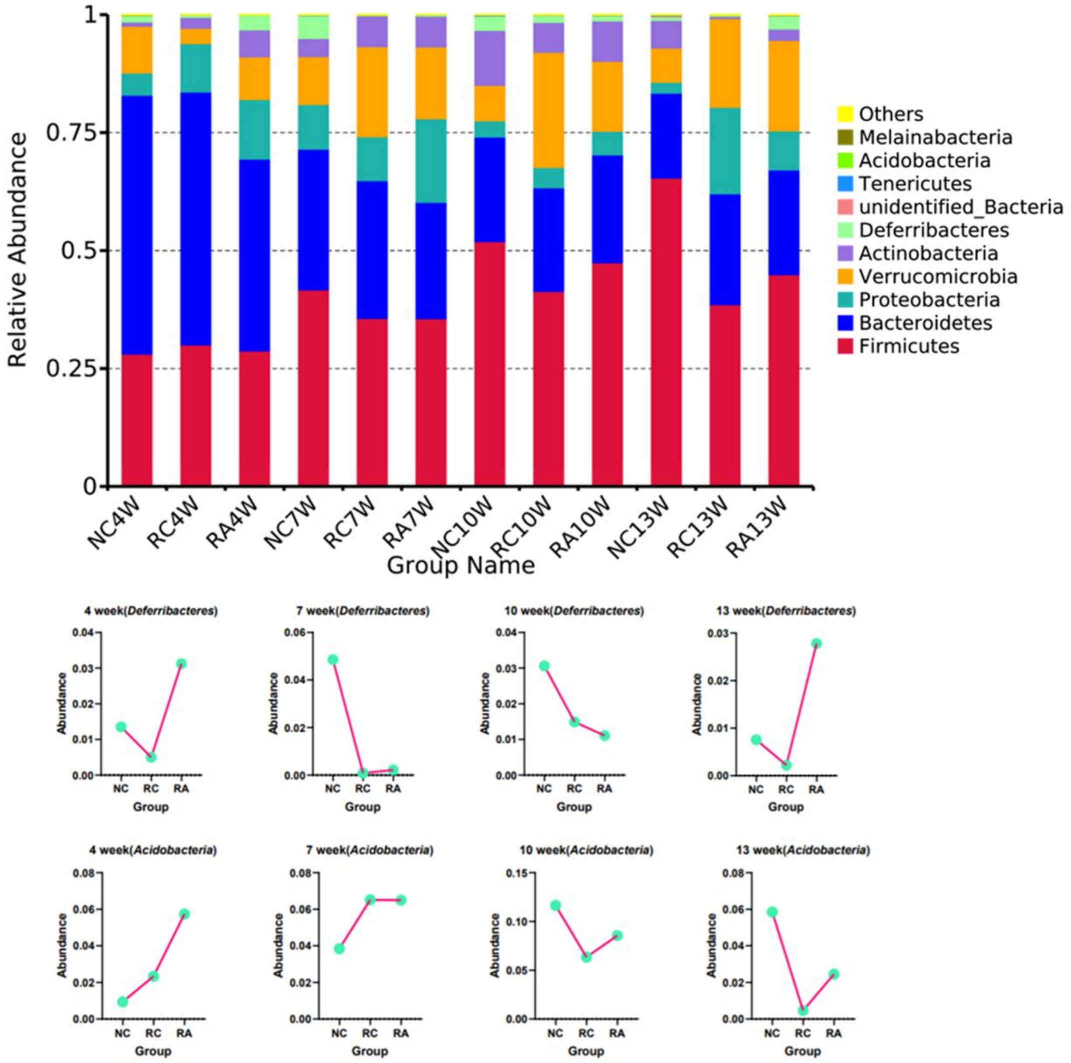

Figure legends

Relative abundance histogram at the phylum level. To determine the relative abundances, the number of tags corresponding to a species in a sample at a certain classification level was divided by the total number of tags corresponding to the OTUs clustered in the sample.

Supplementary Fig. S2

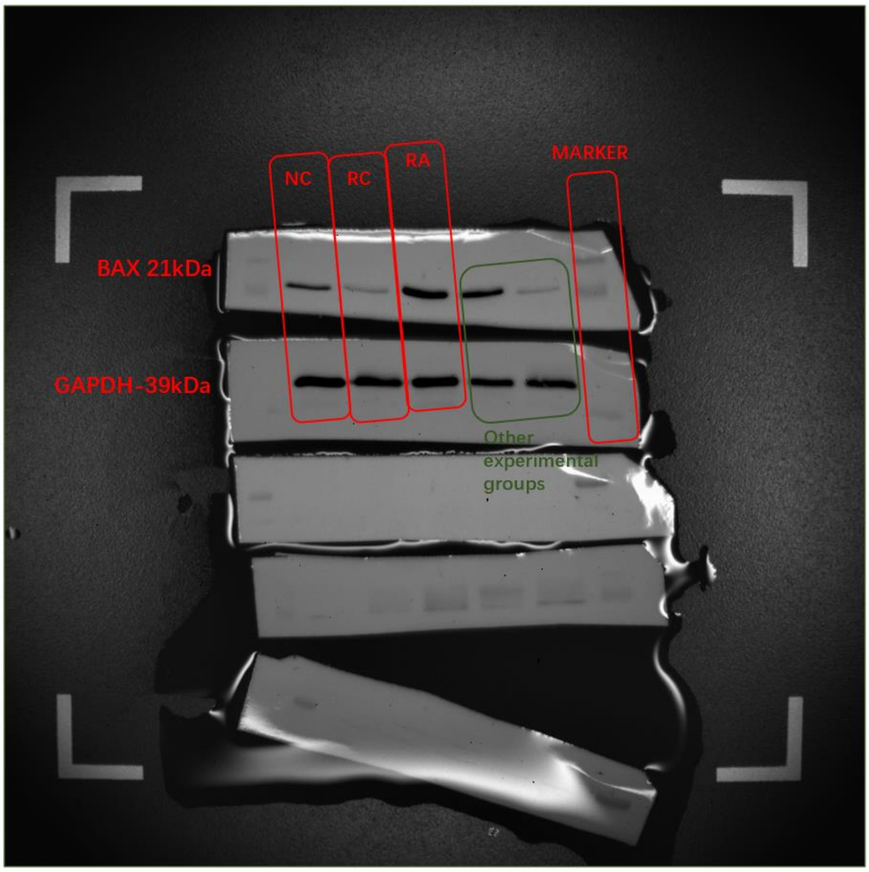

Figure legends

Original blots/gels. Protein was extracted from mouse tumour tissue for Western blotting to determined BAX expression. According to the purpose of the experiment, blots cut prior to hybridisation with antibodies according to the molecular weight of the antibody.We have provided images showing full length membranes, with membrane edges visible in Supplementary Figure S2
